# Important processes during differentiation and early development of somatic embryos of Norway spruce as revealed by changes in global gene expression

**DOI:** 10.1186/1753-6561-5-S7-P78

**Published:** 2011-09-13

**Authors:** Daniel Vestman, Emma Larsson, Daniel Uddenberg, John Cairney, David Clapham, Eva Sundberg, Sara von Arnold

**Affiliations:** 1 Swedish University of Agricultural Sciences, Sweden; 2 NanoBiotechnologies

## Background

Although most morphogenic events in plants occur in the sporophyte following seed germination, the embryonic phase is crucial as it is then that the meristems are specified and the shoot-root body pattern of the plant is established. We are using somatic embryogenesis in Norway spruce (*Picea abies*) as a model system for studying embryology in conifers. A deeper understanding of the genetic regulation can provide clues on how to improve the culture conditions in order to propagate economically important conifers via somatic embryos. The model system includes a well-characterized sequence of developmental stages, resembling zygotic embryogeny, which can be synchronized by specific treatments, making it possible to collect a large number of somatic embryos at specific developmental stages [1]. Here, we have extended the study of early events during embryogenesis in Norway spruce, by using microarray slides spotted with 12,536 cDNA clones from loblolly pine cDNA libraries. We have focused on the first stages of embryogenesis: the differentiation of early embryos from proembryogenic masses (PEMs) and the beginning of the development of late embryos. Few such studies have been undertaken and the work, which we describe here, is the first comprehensive analysis of gene expression of a conifer species during early stages of somatic embryogenesis. The analysis revealed the importance of previously unknown molecular events regulating putative processes associated with pattern formation.

## Methods

The developmental stages of somatic embryogenesis in Norway spruce have been described previously [2] . The stages used in this study included proliferating PEMs on medium containing PGRs, early embryos one week after withdrawal of PGRs and developing late embryos after one week on medium containing ABA.Alteration in gene expression pattern during embryo differentiation and development was analyzed by comparing gene expression of samples from sequential developmental stages. In addition, to identify rapidly occurring transient changes, gene expression in proliferating PEMs was compared with gene expression in PEMs 24 hours after withdrawal of PGRs. Amplified RNA molecules were labeled and expression profiling was conducted using the TIGR loblolly pine microarray [3] with 12,536 validated cDNA clones originating from whole megagametophytes and zygotic and somatic embryos from different stages of development.

## Results

Testing for significant differential expression identified 106, 208 and 464 mRNAs that showed different abundance 24 hours after withdrawal of PGRs, during differentiation of early embryos and development of late embryos, respectively. 53 mRNAs were present in more than one set, giving a total of 720 unique transcripts. The reliability of the microarray results was confirmed by qRT-PCR.

The relative abundances of Gene Ontology (GO) terms within the sets of differentially expressed genes were compared against the GO distribution of all clones on the array.We observed a general over-representation of genes involved in response to stress, both during the transition from proliferation to differentiation of early embryos and from differentiation of early to development of late embryos. During transition from early to late embryogeny, genes involved in catabolic processes, in carbohydrate metabolic processes and in gibberellin (GA) -mediated signaling were over-represented, while genes involved in metabolic processes were under-represented.

Our data show that already 24 hours after withdrawal of PGRs, the elimination of cells by PCD has been initiated as PCD-related genes like *METACASPASE 9* (*MC9*, AT5G04200) were up-regulated.

During differentiation of early somatic embryos, the first week after withdrawal of PGRs, genes related to cell wall modifications were down regulated. An increase in transcript accumulation was observed for genes coding for developmental regulators like MATERNAL EFFECT EMBRYO ARREST 49 (MEE49, AT4G01560), and a decrease of transcripts coding for LOB-DOMAINS containing proteins, indicating that pattern formation has started. The analysis revealed a putative increase in the content of both auxin and gibberellin.

At the developmental switch to late embryogenesis, several genes of importance for cell organization, developmental processes, and other biological processes were up- or down-regulated. The relative transcript abundance of *L1L*, *ROXY1* (AT3G02000) and *WRKY6* (AT1G62300) decreased while the abundance of transcripts such as *ABI3*, *ABI4* (AT2G40220) and *GROWTH-REGULATING FACTOR 1* (*GRF1*, AT2G22840) increased. In addition, genes coding for the LEA proteins were up-regulated. These changes in transcript levels suggest that pattern formation continued and that maturation had initiated at this developmental stage. The transcript levels of genes encoding a fatty acid elongase, FIDDLEHEAD (FDH, AT2G26250), which regulates epidermal cell differentiation, and for an EXTRACELLULAR DERMAL GLYCOPROTEIN (EDGP, AT1G03230) decreased. Transcripts encoding SUR1, MYB77 (AT3G50060), the auxin-induced protein INDOLE-3ACETIC ACID INDUCIBLE 11 (IAA11, AT4G28640), the auxin receptor TRANSPORT INHIBITOR RESPONSE 1 (TIR1, AT3G62980) and a positive regulator of brassinosteroid signalling (AT1G78700) were up-regulated, indicating that the auxin-responsive machinery was up-regulated. Furthermore, genes encoding the GA signaling repressors were up-regulated, while a GA induced and a gene encoding for a GA inactivating enzyme were down-regulated.

## Discussion

By examining changes in global gene expression, we have been able to determine the timing of molecular events regulating putative processes during embryogenesis based on the assumption that the conifer genes are homologous to their Arabidopsis counterparts. We recognize notable changes in the expression of genes involved in regulating auxin biosynthesis and auxin response, gibberellin-mediated signaling, signaling between the embryo and the female gametophyte, tissue specification including the formation of boundary regions, and the switch from embryonic to vegetative development. In addition, our results confirm the involvement of previously described processes, including stress, differentiation of a protoderm and programmed cell death. Our analyses of genes and putative processes that take place during differentiation of early embryos and development of late embryos in a conifer can now serve as a basis for further studies of the processes.

## References

[B1] Bozhkov PV von Arnold S Polyethylene glycol promotes maturation but inhibits further development of Picea abies somatic embryos Physiologia Plantarum 1998 104 211 224 10.1034/j.1399-3054.1998.1040209.x

[B2] Filonova LH Bozhkov PV von Arnold S Developmental pathway of somatic embryogenesis in Picea abies as revealed by time-lapse tracking J Exp Bot 2000 51 249 64 10.1093/jexbot/51.343.249 10938831

[B3] Rensink WA Buell CR Microarray expression profiling resources for plant genomics Trends Plant Sci 2005 10 603 9 10.1016/j.tplants.2005.10.003 16275051

